# Blocking Autophagy Prevents Bortezomib-Induced NF-κB Activation by Reducing I-κBα Degradation in Lymphoma Cells

**DOI:** 10.1371/journal.pone.0032584

**Published:** 2012-02-29

**Authors:** Li Jia, Ganga Gopinathan, Johanna T. Sukumar, John G. Gribben

**Affiliations:** Centre for Haemato-Oncology, Barts Cancer Institute, Barts and The London School of Medicine and Dentistry, Queen Mary University of London, London, United Kingdom; University of Illinois at Chicago, United States of America

## Abstract

Here we show that bortezomib induces effective proteasome inhibition and accumulation of poly-ubiquitinated proteins in diffuse large B-cell lymphoma (DLBCL) cells. This leads to induction of endoplasmic reticulum (ER) stress as demonstrated by accumulation of the protein CHOP, as well as autophagy, as demonstrated by accumulation of LC3-II proteins. Our data suggest that recruitment of both ubiquitinated proteins and LC3-II by p62 directs ubiquitinated proteins, including I-κBα, to the autophagosome. Degradation of I-κBα results in increased NF-κB nuclear translocation and transcription activity. Since bortezomib treatment promoted I-κBα phosphorylation, ubiquitination and degradation, this suggests that the route of I-κBα degradation was not via the ubiquitin-proteasome degradation system. The autophagy inhibitor chloroquine (CQ) significantly inhibited bortezomib-induced I-κBα degradation, increased complex formation with NF-κB and reduced NF-κB nuclear translocation and DNA binding activity. Importantly, the combination of proteasome and autophagy inhibitors showed synergy in killing DLBCL cells. In summary, bortezomib-induced autophagy confers relative DLBCL cell drug resistance by eliminating I-κBα. Inhibition of both autophagy and the proteasome has great potential to kill apoptosis-resistant lymphoma cells.

## Introduction

The proteasome inhibitor, bortezomib, is a novel anti-cancer drug and has been administrated successfully to treat relapsed/refractory multiple myeloma [1], [2]. Previous studies have suggested that proteasome inhibition by bortezomib kills cancer cells via blocking inducible I-κBα degradation and subsequently NF-κB activation [3], [4], [5], or preventing protein degradation of pro-apoptotic proteins such as Bax or p53 [6], [7]. However, it was recently reported that bortezomib-induced accumulation of poly-ubiquitinated proteins leads to formation of aggresomes which minimize their ‘proteotoxicity’ allowing these toxic proteins to be sequestered away from the normal cellular machinery [8], [9], [10].

There are two main routes for eukaryotic intracellular protein clearance: ubiquitin proteasome system (UPS) and autophagy (referred as macroautophagy)-lysosome pathways. The UPS and autophagy degradation systems are functionally coupled and linked by a multi-domain protein adapter, p62,which is able to bind ubiquitinated proteins and lead them to autophagosomes for degradation [11]. It was also found that p62 controls aggresome formation and autophagic degradation [12]. Suppression of the proteasome by bortezomib promotes autophagy in colon cancer cells [13], while inhibition of autophagy increases levels of proteasome substrates, such as p53 protein [14].The search for autophagy client proteins is important to understand how autophagy protects tumor cells from being killed.

NF-κB activation typically relies on two major pathways: canonical and non-canonical. The canonical pathway involves degradation of the NF-κB inhibitor I-κBα, and the non-canonical pathway indicates degradation of NF-κB precursor protein p100. Both I-κBα and p100 proteins were reported to be degraded via UPS [15]. However, a recent study demonstrated that bortezomib induces canonical NF-κB activation rather than inhibition of NF-κB activation by down-regulation of constitutive I-κBα expression in multiple myeloma cells [16]. Others found that treatment of primary effusion lymphoma cells with bortezomib failed to inhibit NF-κB activation [17].

Gene expression profiling in diffuse large B-cell lymphoma (DLBCL) has revealed that this disease has at least three subtypes: germinal centre B-cell like (GCB)-, activated B-cell like (ABC)-and primary mediastinal B-cell lymphoma (PMBL) [18], [19]. Among them, the ABC-DLBCL has higher levels of constitutive NF-κB activity [19]. A previous study showed that DLBCL cells are resistant to treatment with bortezomib alone [20], [21], whereas the combination of bortezomib with other chemotherapeutic drug significantly increased response in ABC-DLBCL compared with GCB-DLBCL [20]. The anti-malaria drug chloroquine (CQ) has been used as an autophagy inhibitor and many studies have shown that CQ strongly potentiates anti-cancer effects of a variety of chemotherapeutic drugs. Treatment with CQ alone induces lymphoma cell death by-passing the mitochondria/caspase-dependent pathway [22]. It is unknown why DLBCL cells are relatively resistant to the proteasome inhibitor bortezomib and whether autophagy plays a role in this resistance.

Our previous study showed that bortezomib kills chronic lymphocytic leukemia cells largely dependent on blocking Bax degradation [6]. In this study, we aimed to determine the resistance factors of DLBCL cells to bortezomib and whether bortezomib induces autophagy during treatment. We demonstrate that bortezomib induces I-κBα degradation which is removed by the autophagic process and activates NF-κB transcriptional activity. Blocking autophagy by CQ potentiates bortezomib-induced accumulation of I-κBα and DLBCL cell death. Taken together, these data suggest a therapeutic role for blockade of this pathway.

## Materials and Methods

### Cells, cell culture and treatment

Primary lymphoma cells were obtained from single cell suspensions of lymph node biopsies after obtaining written informed consent and approval by the East London and the City HA Local Research Ethics Committee 3with REC reference number: 05/Q0605/140 in accordance with the Declaration of Helsinki. DLBCL cell lines used in this study included: the GCB type DoHH2, Su-DHL4, and Su-DHL10and the ABC type Su-DHL8 [23], [24]. Cells were cultured in RPMI 1640 medium supplemented with 10% heat-inactivated fetal calf serum, 25 mM HEPES, and 2.0 mM L-glutamine at 37°C in a 5% CO_2_ humidified incubator.

### Flow cytometry assay cell death and mitochondrial function

Cell death was determined by PI dye exclusion. After treatment, cells were incubated with 10 µg/ml propidium iodide (PI) (Sigma, Poole UK) and the integrity of cell membrane was measured by flow cytometry using a FACS Calibur (Becton Dickinson) on the FL3-H channel. To determine the mitochondrial membrane potential (ΔΨm), after treatment cells was stained with 40 nM Tetramethylrhodamine methyl ester(TMRM) (Invitrogen, Paisley, UK) at 37°C for 15 min and changes in ΔΨm were measured on the FH2-H channel [25]. To measure the production of reactive oxidative species (ROS), cells were stained with 40 µM dihydroethidium (Polyscience-Park Scientific Ltd, Northampton, UK) at 37°C for 15 min and increases in ROS production were measured on the FH3-H channel [25].

### Drug uptake

Cells (2×10^5^/ml) were subjected to flow cytometry for 10 seconds in pre-warmed culture medium to establish a base-line of cellular auto-fluorescence. The assay was interrupted, and daunorubicin (100 µg/ml) was added. Red fluorescence was measured continuously at a flow rate of 200 cells per second for 100 seconds on a log scale [26].

### Proteasome and caspase activity assays

Cytosolic proteins (50 µg) were diluted to 90 µl with fluorogenic assay buffer (20 mM HEPES-KOH, pH 7.4, 10 mM DTT, 10% sucrose, 1.0 mM EDTA, 0.1% CHAPS). The reaction was initiated by addition of 10 µl of 400 µM (final concentration was 40 µM) fluorescent substrates, Suc-LLVY-AMC (Enzo Life Science, Exeter, UK) for the chymotrypsin-like peptidase activity of the 20 S proteasome or Ac-DEVE-AFC (Merck-Calbiochem, Nottingham, UK) for the caspase-3 activity. The cleavage reaction was carried out at 37°C for 15 min. The fluorescence at 400/505 nm for caspase-3 or at 380/460 nm for proteasome was measured with a BMG LABTECH POLARstar OPTIMA Microplate Reader (Offenburg, Germany). Measurements were calibrated against a standard linear regression curve of AFC or AMC. Caspase or proteasome activity was defined as µM AFC or AMC release per mg protein per hour (µM/hr/mg protein) [6].

### Preparation of cytosol and nuclear protein extracts

Cytosolic or nuclear proteins were extracted using the Nuclear Complex Co-IP Kit (Active Motif Europe, Rixensart, Belgium) according to the manufacturer's instruction. Briefly, cells were swelled by incubation with hypotonic buffer containing protease inhibitor or phosphatase inhibitor cocktails at 4°C for 15 min and then mixed with detergent solution (1∶20 dilution). Cytosol and nucleus were separated by centrifugation at 14,000×*g* and nuclear fraction was washed once with the hypotonic buffer. The nuclear proteins were extracted with the Complete Digestion Buffer. The purities of cytosolic and nuclear proteins were examined by Western blotting using LDH for cytosol and Rb antibody for nuclear fractions.

### Co-immuno-precipitation (Co-IP)

Co-IP was performed using the Nuclear Complex Co-IP Kit and Dynabeads protein A (Invitrogen). Dynabeads in 50 µl of IP buffer was incubated with 2 µg anti-IκB-α antibody or rabbit IgG for 10 min at room temperature on a rotator. After three washes with IP buffer, Dynabeads protein A coated with antibody or IgG was mixed with 300 µg proteins and incubated for 20 min at room temperature on a rotator. Protein complex was then eluted with NuPAGE LDS sample buffer mixed with reducing agent (Invitrogen).

### Measurement of NF-κB p65 DNA binding activity

Nuclear proteins (10 µg) were used to determine DNA binding activity of NF-κB p65 using TransAM NF-κB p65 kit (Active Motif). NF-κB p65 bound to the immobilized oligonucleotide containing a p65 binding site was detected by ELISA with colorimetric reagent.

### Determine NF-κB DNA binding activity by Electrophoretic Mobility Shift Assay

NF-κB DNA binding activity was carried out by electrophoretic mobility shift assay (EMSA). This assay is based on the fact that DNA-protein complexes migrate slower than non-bound DNA in native polyacrylamide, resulting in a “shift” in migration of the labeled DNA band. Nuclear protein-DNA complexes were extracted by NE-PER® Nuclear and Cytoplasmic Extraction Reagents (Pierce-Thermo Scientific). Double stranded NF-κB consensus oligonucleotides 5′-AGT TGA GGG GAC TTT CCC AGG C-3 and 3′-TCA ACT CCC CTG AAA GGG TCC G-5′ (Santa Cruz) containing a putative binding site for NF-κB were end-labeled with biotin using Biotin 3′ End DNA Labeling Kit (Pierce-Thermo Scientific). Bands were detected by “The Light Shift™ Chemiluminescent EMSA kit” (Pierce-Thermo Scientific) using a non-isotopic method to detect DNA-protein interactions. Biotin end-labeled DNA duplex of NF-κB was incubated with the nuclear extracts (10 µg nuclear proteins). After the reaction the DNA-protein complexes were subjected to a 6% DNA retardation gel (Invitrogen) and transferred to a positively charged nylon membrane (Pierce-Thermo Scientific). After transfer the membrane was immediately cross-linked for 15 min on a Gene Flash UV transilluminator (Syngene). A chemiluminescent detection method utilizing a luminol/enhancer solution and a stable peroxide solution (Pierce-Thermo Scientific) was used as described by the manufacturer, and membranes were exposed under Luminescent Image Analyzer (FujiFilm) for visualization.

### Western blotting

Proteins were extracted with CelLytic™ M cell Lysis Reagent (Sigma) supplied with protease inhibitor and phosphatase inhibitor cocktails (Sigma). Protein concentration was determined by Bradford method, using Bio-Rad Bradford reagent. Proteins were mixed with NuPAGE LDS Sample Buffer (Invitrogen) and boiled for 5 min before analysis by Western blotting. Proteins were subjected to 4–20% NuPAGE gels (Invitrogen) and transferred onto PVDF membrane (Sigma) at 20 V for 1 hour by semi-dry transfer. PVDF membrane was blocked with 5% non-fat milk in PBST or TBST for 1 hour and then incubated with primary antibodies overnight at 4°C against the following: Bcl-2 (100), NF-κB p65 (F-6), ubiquitin (P4D1), Bcl-x_S/L_ (S-18), I-κBα (FL), LDH (H-160), and PARP (H-250) (Santa Cruz); β-actin (AC-74), FLIP and p62/SQSTM1 (Sigma); IL-6 (R&D Systems); CHOP, phospho-IκBα-(Ser32/36), Rb, LC3B, phospho-NF-κB p65-(Ser536), and Mcl-1, NF-κB p65 (RelA), RelB, c-Rel,and NF-κB p50 antibodies (Cell Signaling Technology-New England Biolabs, Hitchin, UK). Bound antibodies were detected using appropriate HRP-conjugated secondary antibodies (Santa Cruz), visualized by GeneSnap (SynGene, Cambridge, UK) after adding ECL plus (GE Healthcare Life Science), and the density of each band was analyzed with the Gelscan V5.1 software.

### Immuno-staining and fluorescent microscopy

Cells were fixed and permeabilized on slides with Cytofix/Cytoperm reagents (BD) and blocked with a buffer consisting of 0.1% saponin and 5% serum (the type of serum corresponding to the isotype of the secondary antibody). Cells were co-stained with p62 antibody (mouse 1∶20 or rabbit 1∶150), monoclonal anti-ubiquitin antibody (1∶20), polyclonal anti-LC3 antibody (1∶100), or polyclonal anti-I-κBα (1∶20) for 1 hour at room temperature. After washing with PBST, cells were incubated with Alexa-Fluor conjugated secondary antibodies at a 1∶100 dilution for Alexa-Fluor 488 and 1∶50 dilution for Alexa-Fluor 546 (Invitrogen). Slides were washed for 3 times with PBST, stained with 50 ng/ml DAPI (4′,6-diamidino-2-phenylindole), air-dried at 4°C in the dark, and mounted in ProLong® Gold anti-fade reagent (Invitrogen) before being viewed under an Olympus BX40 fluorescence microscope (Artisan Scientific Corporation, USA).

### Transfection of plasmid

Plasmid DNA was extracted using Plasmid DNA extraction kit (Sigma). Transfection was performed using Nucleofector™ II device and Nucleofector Reagent C (Lonza Biologics, Slough, UK). Su-DHL8 cells (5×10^6^/ml) in the Reagent C were mixed 1 µg pBABE-puro mCherry-EGFP-LC3B construct (Addgene) and the program D-023 was used for the transfection.

### Statistical analysis

Data are presented as means ± standard deviation (SD) and were analyzed by build-in *t* test, Correlation and ANOVA with replications using Microsoft Excel and Prism software. Change where P≤0.05 was considered significant. The synergistic effect of chloroquine (CQ) on bortezomib-induced cell death was analyzed by the CalcuSyn software. In this analysis, the general range of CI values of less than 0.90 indicate various levels of synergy, while values of 0.90–1.10 indicate addition, and >1.10 indicates antagonism [27].

## Results

### Bortezomib-induced proteasome inhibition mediates autophagy

**Figure 1:**
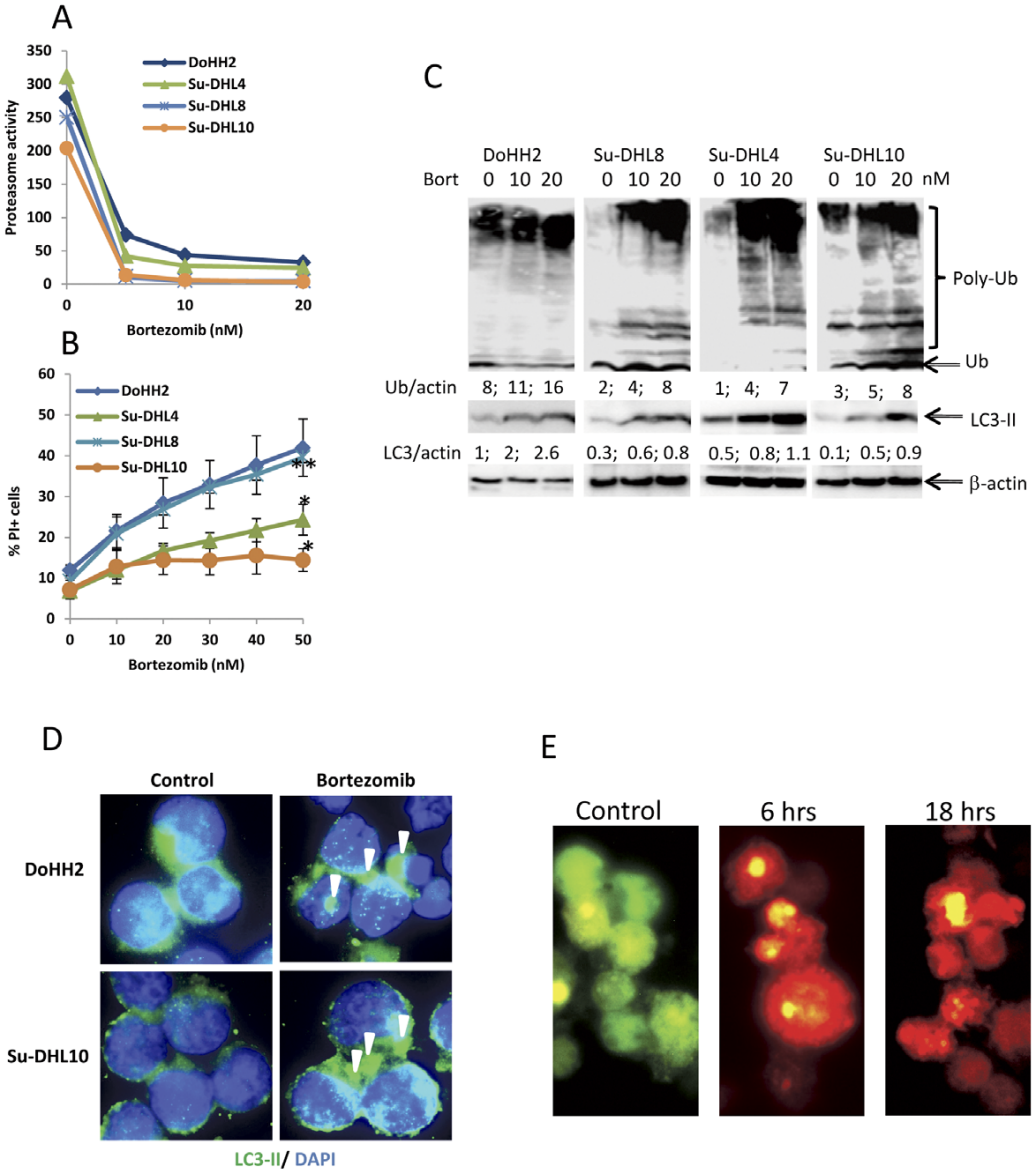
Bortezomib-induced proteasome inhibition and autophagy. (A) Bortezomib-induced proteasome inhibition. Proteasome activity was measured by fluorogenic assay after cells were treated with bortezomib (Millennium) for 16 hours. Error bars are omitted for clarity. (B) Bortezomib-induced cell death. Cell death was determined by flow cytometry of PI positive cells (PI^+^) after 24 hours treatment. Data presented (A and B) are mean ± SD from three independent experiments. Significant difference in cell death was observed by ANOVA with replicated data between DoHH2 and SU-DHL4 and Su-DHL10 (*P<0.0001) whereas no difference was observed between DoHH2 and Su-DHL8 (**P>0.05). (C) Bortezomib-induced accumulation of poly-ubiquitinated proteins and autophagy protein LC3-II. Cells were treated with bortezomib for 24 hours (DoHH2 and Su-DHL8) or 48 hours (Su-DHL4 and Su-DHL10). Cell pellets were lysed with 1% SDS containing lysis buffer. After heated at 99°C for 10 min, proteins were cleared by ultracentrifugation. Monoclonal anti-ubiquitin antibody was used at 1∶200 dilution. Polyclonal anti-LC3B antibody was used at 1∶1000 dilution and β-actin was used as a loading control. Numbers under each group of Western blots are ratios of ubiquitin/β-actin or LC3-II/β-actin which were measured by densitometry. (D) Autophagosome formation. After treatment with 20 nM bortezomib for 24 hours, fixed and permeabilized cells on slides were stained with the anti-LC3B antibody (1∶100 dilution) and then anti-rabbit Alexa Fluor-488 (1∶100 dilution). DAPI was used to indicate nuclear localization. Arrow heads indicate autophagosomes. (E) Formation of autophagosomes and autolysosomes. Cells were transfected with 1 µg pBABE-puro mCherry-EGFP-LC3B plasmids for 48 hours and then treated with 20 nM bortezomib for 24 hours.

In keeping with previous studies [20], [21], we found that cells obtained from lymph node biopsies of previously untreated DLBCL patients were resistant to bortezomib-mediated killing. To understand the mechanism for this, we assessed the sensitivity of lymphoma cell lines to bortezomib-induced proteasome inhibition and cell death. These cell lines showed equal capacity of drug uptake (Figure S1). Bortezomib at the concentration of 10 nM reached almost maximum inhibition on the proteasome activity in these cell lines (Figure 1A). In agreement with a previous study [28], the sensitivity of these DLBCL cell lines to bortezomib-induced cell death was not associated with proteasome inhibition (Figure 1B). The ABC-DLBCL cell line Su-DHL8 showed a similar sensitivity to bortezomib-induced cell death compared with the GCB-DLBCL cell line DoHH2 (Figure 1B).

Accumulation of poly-ubiquitinated proteins as a result of bortezomib-induced proteasome inhibition was detected by Western blotting (Figure 1C). The UPS and autophagy systems are functionally linked and inhibition of UPS stimulates autophagic activity, possibly as a compensatory mechanism [29], [30]. We therefore tested whether bortezomib-induced accumulation of ubiquitinated proteins triggers autophagy in DLBCL cells. Bortezomib induced a dose-dependent LC3-II accumulation, a hallmark of autophagy activation, in both sensitive and resistant cell lines but LC3-I was neither detected in control cells nor in bortezomib-treated DLBCL cells (Figure 1C). The amounts of LC3-II accumulation were strongly correlated to those of accumulated poly-ubiquitin (Correlation P<0.0001, Figure S2). To further confirm the role of bortezomib on the induction of autophagy, we determined the LC3-II aggregation by immunostaining on cells treated with bortezomib. Larger LC3-II aggregates were formed after treatment with bortezomib in both the bortezomib-sensitive DoHH2 and resistant Su-DHL10 cell lines, demonstrating the formation of autophagosomes (Figure 1D). Autophagosome formation was also confirmed in mCherry-EGFP-LC3B transfected Su-DHL8 cells. Because EGFP is acid-labile and mCherry is acid-stable, transfection of the mCherry-EGFP-LC3B construct allows visualization of both autophagosomes (neutral) and autolysosomes (acidic) [31]. After treatment with bortezomib for 6 or 18 hours, most of the cells contained red colored structures, indicating autolysosomes (Figure 1E). These results demonstrate that treatment of DLBCL cells with bortezomib induces autophagy which enables cells to remove accumulated proteins.

### Bortezomib induces cellular stress responses

**Figure 2:**
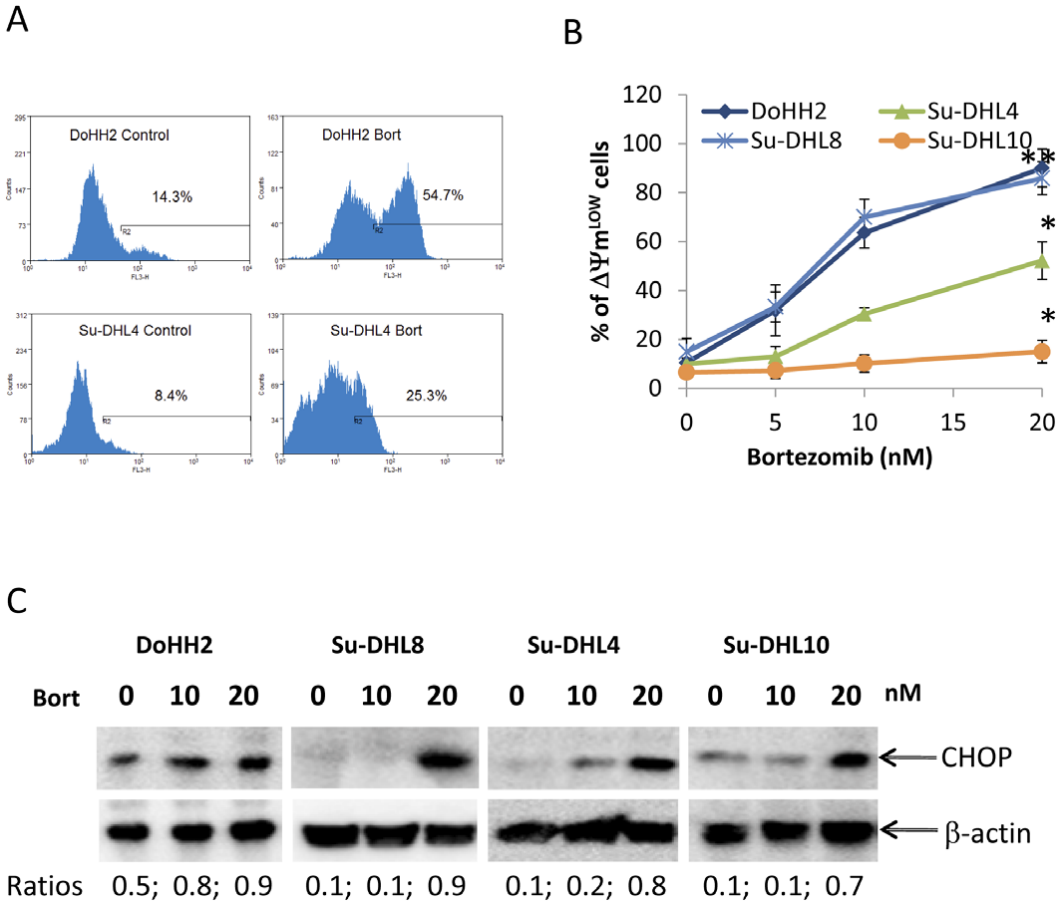
Bortezomib-induced cellular stress responses. Bortezomib-induced ROS generation (A) and mitochondrial depolarization (B). Cells were treated with 20 nM (A) or different concentrations of bortezomib for 18 hours (DoHH2 and Su-DHL8) or 24 hours (Su-DHL4 and Su-DHL10). ROS or ΔΨm was determined by flow cytometry after cells were stained with HE or a ΔΨm-dependent dye TMRM, respectively. (A) A typical result from three independent experiments performed. (B) Data presented are mean ± SD from three independent experiments. By comparison with the DoHH2 cell line, significant differences by ANOVA were observed for Su-DHL4 and Su-DHL10 (*P<0.001), whereas no significant difference was observed for Su-DHL8 (**P>0.05). (C) Bortezomib-induced accumulation of CHOP was determined by Western blotting using anti-CHOP antibody at 1∶1000 dilution.

Inhibition of proteasome activity causes accumulation of ubiquitinated and/or mis-folded proteins. This could lead to cellular stress including mitochondrial and ER stress, which in turn triggers either cell death and/or the autophagic process [6], . We therefore determined whether treatment with bortezomib induces these cellular stress responses in DLBCL cell lines and whether these responses are pro- or anti-apoptotic reactions. Cellular stress after treatment with bortezomib was firstly observed at the mitochondrial levels, as increased generation of ROS (Figure 2A) and decreased ΔΨm (Figure 2B). DLBCL cells showed similar sensitivity patterns to bortezomib-mediated mitochondrial depolarization (Figure 2B) and cell death (Figure 1B), suggesting that bortezomib-induced cytotoxicity is via the mitochondria-dependent pathway. Perturbations of ER homeostasis by proteasome inhibition affects protein folding and causes accumulation of CHOP protein, an indicator of ER stress. It was reported that accumulation of CHOP could mediate ER stress-associated apoptosis [32].Bortezomib induced a dose-dependent accumulation of CHOP protein (Figure 2C), the levels of which were not associated with the sensitivity of bortezomib-induced cell death (Figure 2C and Figure 1B). These data suggest that bortezomib-induced cell death was not caused by ER stress.

### p62 recruits ubiquitinated proteins including I-κBα to autophagosomes

**Figure 3:**
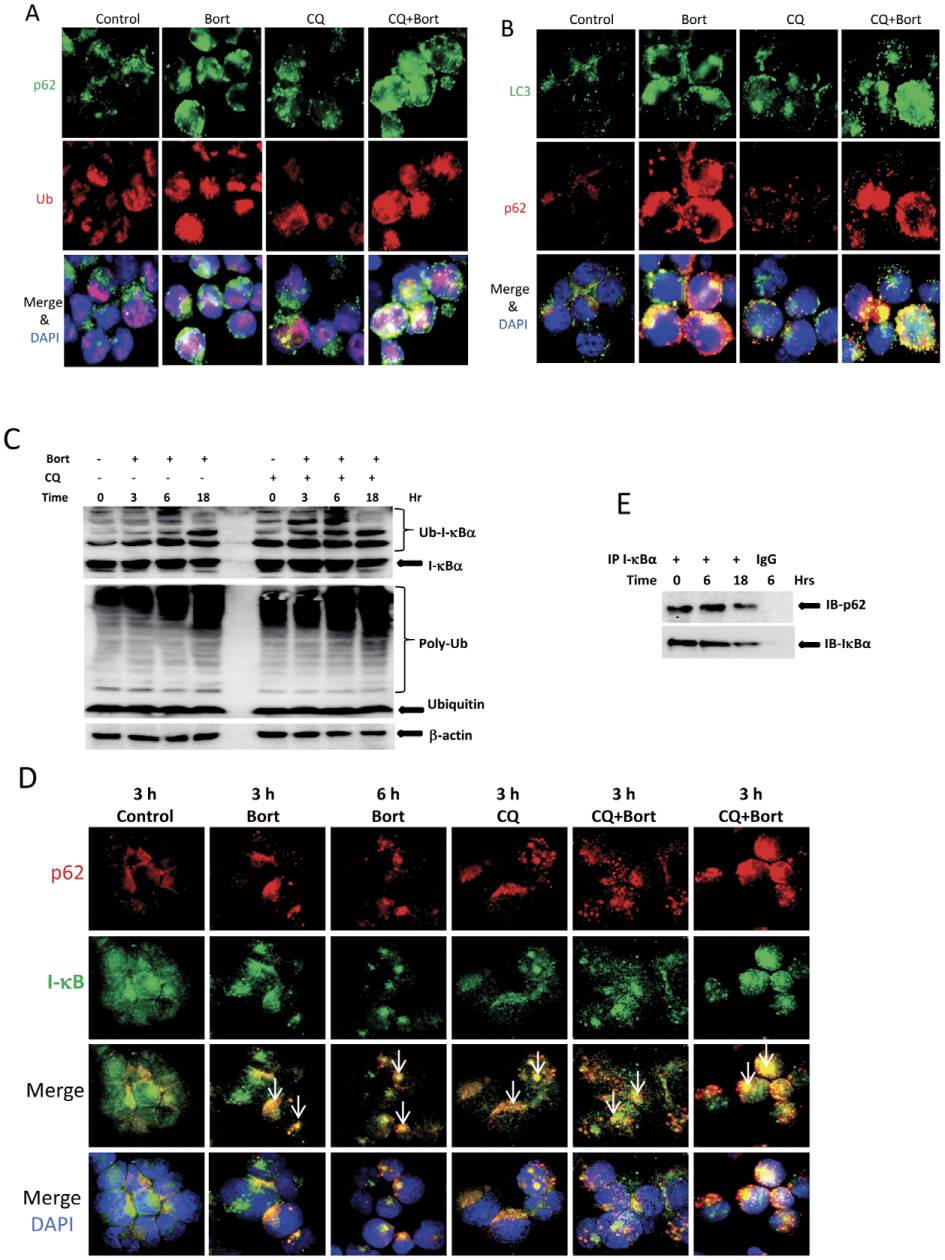
Role of p62 on sequestration of ubiquitin and I-κBα. Su-DHL8 cells were pre-incubated with or without 50 µM CQ for 1 hour and then treated with indicated concentration of bortezomib for 24 hours. (A) Co-localization of ubiquitin and p62. Cells on slides were co-stained with monoclonal anti-ubiquitin (1∶20 dilution) and polyclonal anti-p62 (1∶100 dilution) antibodies, and probed with Alexa Fluor anti-mouse IgG 546 (1∶50 dilution) and anti-rabbit IgG-488 (1∶100 dilution) antibodies. (B) Co-localization of p62 and LC3-II. Cells on slides were co-stained with monoclonal anti-p62 (1∶20 dilution) and polyclonal anti-LC3B (1∶100 dilution), and probed with Alexa Fluor anti-mouse IgG 546 (1∶50 dilution) and anti-rabbit IgG-488 (1∶100 dilution) antibodies. (C) Bortezomib-induced ubiquitination of I-κBα and accumulation of LC3-II protein. After treatment with 20 nM bortezomib with or without CQ, proteins were extracted from Su-DHL8 cells with 1% SDS and loaded onto 4–20% NuPAGE. Polyclonal anti-IκBα (1∶200) and monoclonal anti-ubiquitin (1∶200) were used to probe specific proteins. (D) Co-localization of p62 and I-κBα. Cells on slides were co-stained with polyclonal anti-I-κBα antibody (1∶20) and monoclonal anti-p62 antibody (1∶20) and then probed with Alexa Fluor anti-mouse IgG 546 (1∶50 dilution) and anti-rabbit IgG-488 (1∶100 dilution). Arrows indicate p62-I-κBα aggregates. (E) Detection of p62 and I-κBα interaction by Co-IP. Polyclonal anti-I-κBα antibody (2 µg) or normal rabbit IgG was coated onto Dynabeads protein A and 300 µg proteins were used for IP. Monoclonal anti-p62 antibody (1∶100 dilution) and polyclonal anti-I-κBα antibody were used to detect p62/I-κBα protein complex.

When the proteasome is inhibited, ubiquitinated proteins can also be digested by autophagy [14]. The adaptor protein p62 recruits ubiquitin and its conjugated proteins, leading them to the autophagosome by also interacting with the autophagy protein LC3 [33], [34]. Immunostaining experiments showed that ubiquitin (red) and p62 (green) were separately localized in untreated Su-DHL4 cells. After treatment with 10 nM bortezomib for 24 hours, ubiquitin was recruited by p62, shown by co-localization of red (ubiquitin) and green (p62) merged into large, dense, yellow aggregates. Accumulation of ubiquitin and p62 aggregates increased in both number and size when cells were treated with both CQ and bortezomib (Figure 3A). Similar results were observed in the other cell lines (Figure S3 and results not shown). To determine whether p62 directed protein aggregates to autophagosomes, cells were co-stained with p62 (red) and autophagosome protein LC3 (green). In the control cells, the patterns of both LC3 and p62 distribution in the cytosol were either as smeared or in small dots. Treatment with either bortezomib or CQ alone increased formation of autophagosomes, shown as increased LC3 granules. Combination of both CQ and bortezomib showed stronger accumulation of LC3 and p62 aggregates (Figure 3B and Figure S4).

I-κBα is a short-lived protein, previously thought to rely on UPS for degradation [35]. However, it was recently found that I-κBα protein degraded in multiple myeloma cells and some cancer cell lines during treatment with bortezomib [16], [36]. We show that uiquitination of I-κBα was detected in Su-DHL8 cells in the resting stage and increased after treatment with bortezomib or/and CQ for 3 to 6 hours. Bortezomib-induced accumulation of poly-ubiquitinated proteins increased when combined with the autophagy inhibitor CQ (Figure 3C). We therefore tested whether bortezomib-induced I-κBα degradation also occurs in DLBCL cells and whether this degradation is associated with enhanced autophagy. To test whether I-κBα degradation by autophagy is associated with p62 aggregation, DLBCL cells were co-stained with p62 (red) and I-κBα (green) after treatment with CQ and 20 nM bortezomib up to 24 hours. I-κBα and p62 co-localization was observed in both control and bortezomib-treated cells for 3, 6 and 24 hours, shown as larger yellow speckles, although the intensity of I-κBα green fluorescence was reduced in bortezomib-treated cells. Accumulation of I-κBα/p62 aggregates was intensified when cells were treated with both CQ and bortezomib (Figure 3D and Figure S5), indicating blocking autophagy prevented I-κBα and p62 degradation. These results demonstrate that p62 recruits ubiquitinated proteins, such as I-κBα, and leads them to autophagosomes for digestion. On immunoprecipitation with I-κBα antibody, interaction between I-κBα and p62 was detected in both the resting stage and after treatment with bortezomib (Figure 3E). These results demonstrate that p62 could be a carrier to bring ubiquitinated I-κBα to autophagosomes for degradation.

### Inhibition of autophagy by CQ prevents bortezomib-induced I-κBα degradation but not phosphorylation

**Figure 4:**
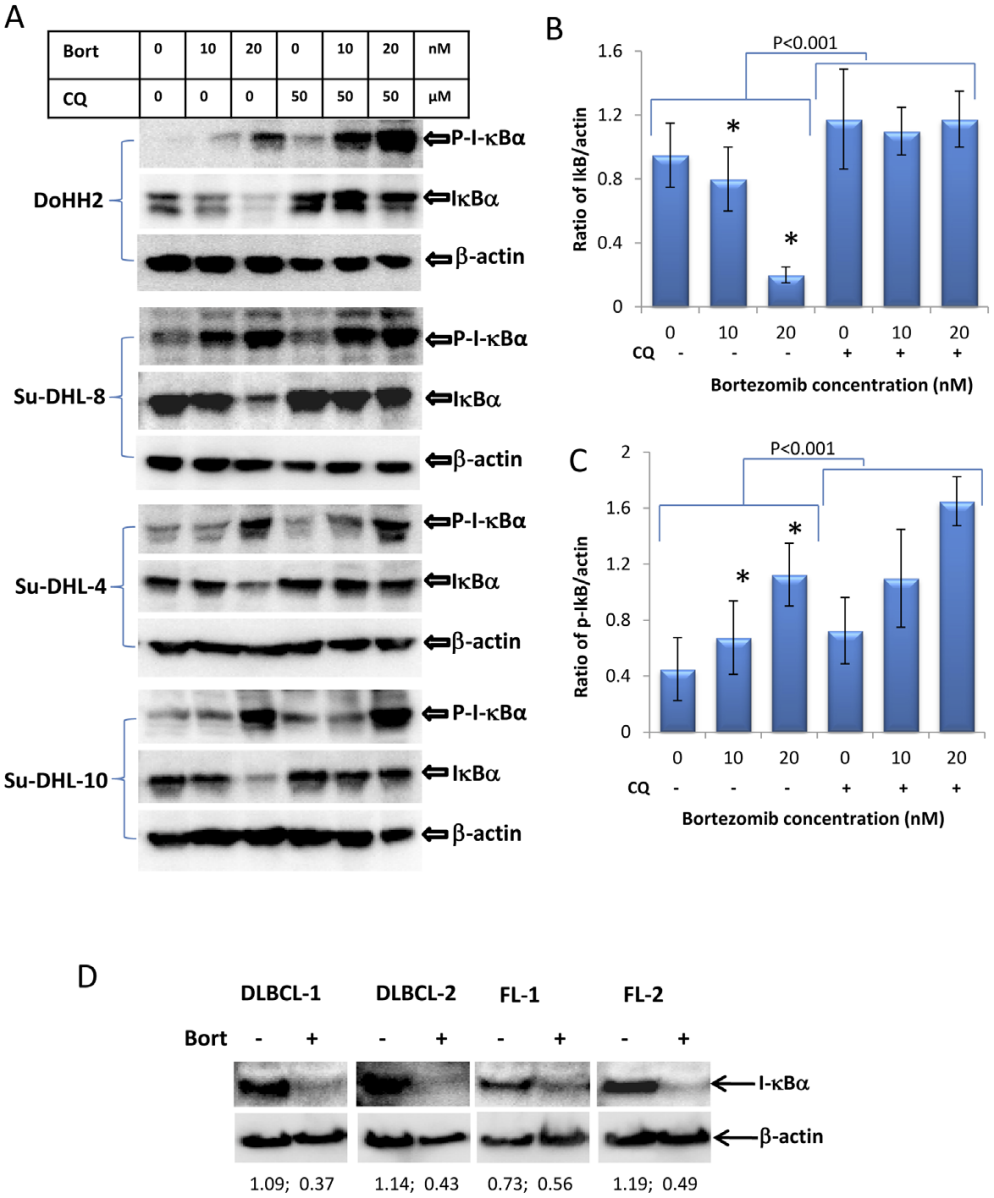
Bortezomib-induced I-κBα phosphorylation and degradation. DoHH2, Su-DHL4, Su-DHL8 and Su-DHL10 cell lines were pre-treated with 50 µM CQ for 1 hour and then treated with 10 nM or 20 nM bortezomib for 24 hours. Proteins were extracted in the presence of phosphatase inhibitor cocktails. Polyclonal anti-phospho-I-κBα (Ser32/36) (1∶1000) and anti-I-κBα (1∶200) antibodies were used for Western blotting. Ratios of I-κBα/β-actin (B) and P-I-κBα/β-actin (C) were analyzed by densitometry. Data represent mean ± SD from the four cell lines tested (*P<0.001). (D) Bortezomib-induced I-κBα degradation in primary lymphoma samples. Single suspension cells were treated with 20 nM bortezomib for 24 hours and proteins were extracted for Western blotting. Polyclonal anti-I-κBα antibody was used at 1∶200 dilution.

NF-κB activation is initiated by phosphorylation and consequently degradation of its inhibitor protein I-κBα. Bortezomib induced dose-dependent I-κBα degradation was coupled with increased phosphorylation, as detected by Western blotting in four DLBCL cell lines. For the 4 cell lines tested bortezomib-induced I-κBα degradation was significantly (p<0.01) inhibited by pre-treatment with the autophagy inhibitor CQ (Figure 4A and 4B). Inhibition of autophagy did not block bortezomib-mediated phosphorylation of I-κBα. In contrast, the levels of phosphorylated I-κBα were significantly (p<0.01) increased (Figure 4A and 4C). This indicates that the role of CQ is to prevent I-κBα degradation by autophagy but not upstream of its phosphorylation. Bortezomib-induced I-κBα degradation was also observed in primary FL and DLBCL cells (Figure 4D).

### Inhibition of autophagy blocks bortezomib-induced NF-κB activation

**Figure 5:**
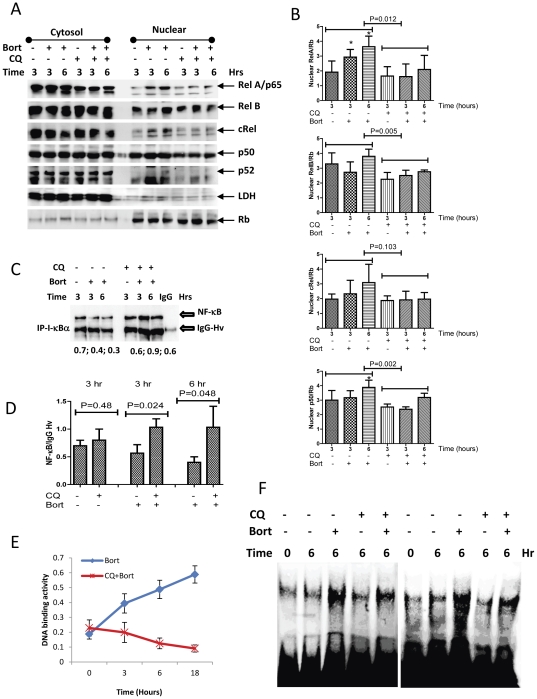
Bortezomib-induced NF-κB activation. Su-DHL8 cells were treated with 20 nM bortezomib with or without 50 µg/ml of CQ. (A and B) Effects of bortezomib or/and CQ on the levels of NF-κB proteins. Cytosolic and nuclear proteins were extracted from Su-DHL8 cells. 15 µg cytosolic or nuclear proteins were subjected to 4–20% NuPAGE gel. Anti-NF-κB RelA/p65, RelB, cRel, p50 and p52 rabbit antibodies were used at 1∶3000 dilution. Monoclonal Rb (the nuclear protein marker), and polyclonal LDH (the cytosolic protein marker) antibodies were used at 1∶2000 and 1∶500 dilution, respectively. Western blots presented are representative of 3 independent experiments performed. (B) Data analysis of NF-κB nuclear translocation. Significantly increased p65 or p50 protein levels after treatment with bortezomib (*P<0.05) was compared with their controls and analysed by t test (n = 3). Chloroquine-mediated inhibition on the nuclear translocation of RelA/p65, RelB, cRel and p50 was compared with their controls and treated with bortezomib alone. Significantly decreased nuclear protein levels were analyzed by t-test. (C) Effects of bortezomib or/and CQ on interaction between NF-κB and I-κBα. Dynabeads proteins A was coated with 2 µg of polyclonal anti-I-κBα antibody or normal rabbit IgG and then incubated with 300 µg protein. Interaction of I-κBα with NF-κB was determined by Western blotting with monoclonal anti-NF-κB antibody. IgG-Hv indicates IgG heavy chain (55 kD). Numbers under each pairs of bands are ratios of NF-κB/heavy chain. (D) Statistical data from three independent IP experiments analyzed by t test. (E) Effects of bortezomib or/and CQ on NF-κB RelA/65 DNA binding activity. Nuclear proteins were extracted using Nuclear Co-IP Kit. 10 µg nuclear proteins were used for the DNA binding assay. NF-κB DNA binding activity was determined using TransAM™ NF-κB p65 ELISA Kit. Data represent mean ± SD from 3 independent experiments. (F) Effects of bortezomib or/and CQ on NF-κB/cRel DNA binding activity. Su-DHL8 cells were treated with 20 nM bortezomib with or without 50 µM CQ for 6 hours. Protein-DNA complexes were extracted and bound with biotin-labeled NF-κB/cRel DNA binding site. Data represent two separate results from four independent experiments.

We next determined whether bortezomib induces NF-κB activation. The ABC-DLBCL cell line Su-DHL8 showed constitutive NF-κB activity, as detected by nuclear localization of NF-κB family proteins, mainly RelB and p50. Treatment with bortezomib significantly increased NF-κB family proteins RelA/p65 and p50 nuclear translocation. Modestly increased RelB and c-Rel nuclear translocation were also observed after treatment with bortezomib. However, p52 nuclear translocation was not detected. Blocking autophagy by CQ significantly decreased RelA/p65, RelB, c-Rel and p50 nuclear protein levels (Figure 5A and 5B). Immuno-precipitation results showed that bortezomib reduced the interaction between NF-κB RelA/p65 and I-κBα and co-treatment with CQ prevented bortezomib-induced disruption of NF-κB and I-κBα binding (Figure 5C and 5D). Consistent with the co-immuno-precipitation results, bortezomib remarkably increased NF-κB RelA/p65 DNA binding activity. Importantly, treatment with CQ blocked bortezomib-induced DNA binding activity (Figure 5E). Bortezomib-mediated NF-κB activation was also confirmed by electrophoretic mobility shift assay. After treatment for 6 hours, NF-κB activity was remarkably enhanced bortezomib. Bortezomib-induced NF-κB activation was also reduced by co-treatment with CQ (Figure 3F). Taken together, these results demonstrate that bortezomib induces canonical NF-κB activation by triggering the autophagic degradation of I-κBα.

### Bortezomib enhances *de novo* protein synthesis of NF-κB target genes

**Figure 6:**
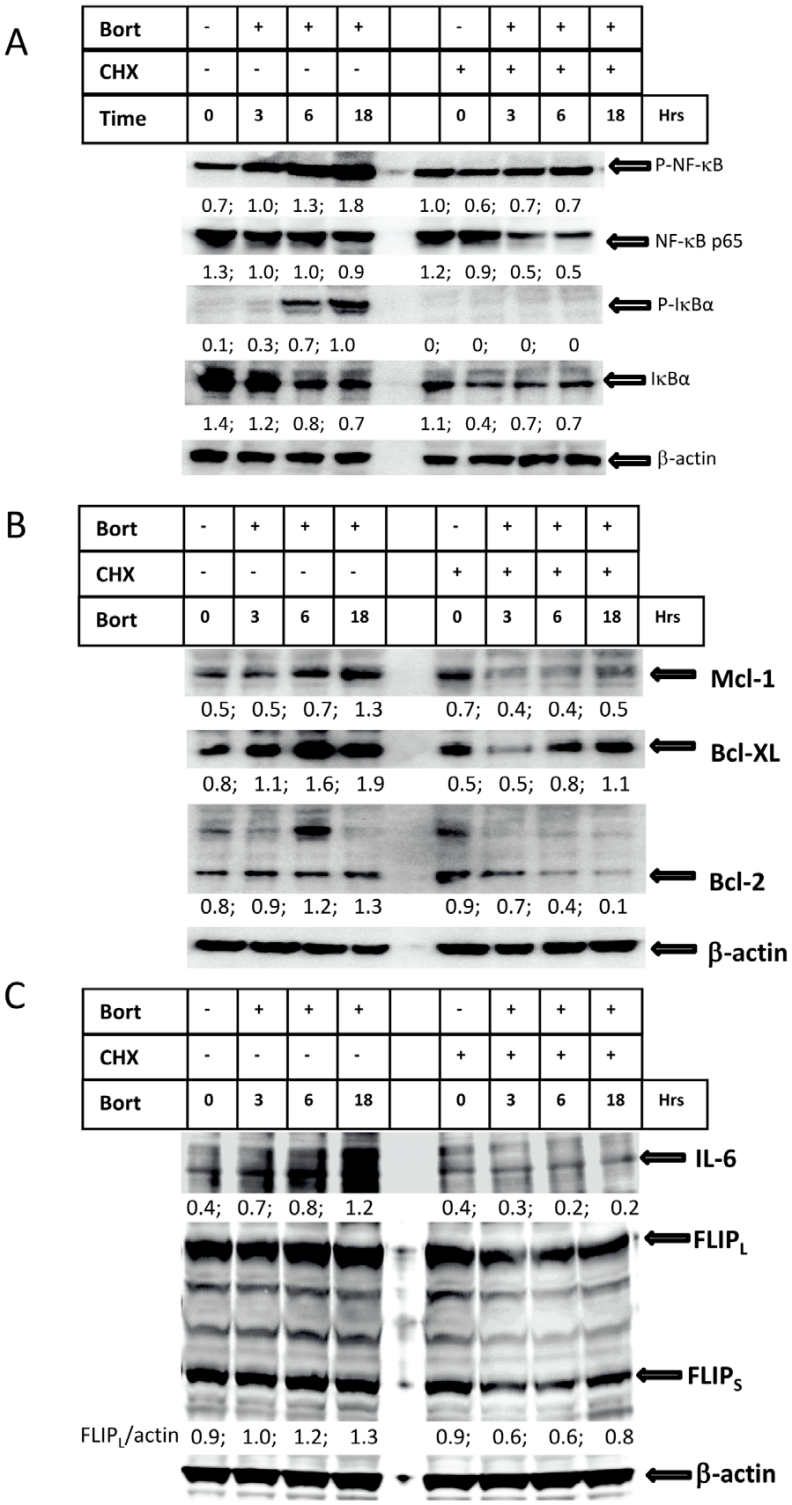
Bortezomib-induced protein synthesis. Su-DHL-8 cells were pre-treated with 50 µg/ml CHX for 1 hour and then incubated with 20 nM bortezomib for indicated period of time. (A) Effect of CHX on NF-κB and I-κBα. Monoclonal anti-NF-κB (1∶200) and anti-phospho-I-κBα (1∶1000), and polyclonal anti-phospho-NF-κB (1∶1000) and I-κBα were used for the Western blotting. (B) Effect of CHX on anti-apoptotic proteins. Polyclonal anti-Mcl-1 (1∶1000) and Bcl-XL (1∶200) antibodies, and monoclonal anti-Bcl-2 antibody (1∶200) were used for Western blotting. (C) Effect of CHX on other NF-κB target proteins. Goat anti-IL-6 (1∶1000) and rabbit anti-FLIP (1∶2000) antibodies were used for Western blotting. Numbers on the bottom of each lane are ratios of indicated protein to β-actin.

We next determined whether bortezomib-mediated activation of NF-κB triggers protein synthesis of its target genes. NF-κB itself and its inhibitor I-κBα are among NF-κB target genes [37]. We detected that treatment with bortezomib induced increased protein expression of the serine-536 phosphorylated, but not non-phosphorylated, form of NF-κB p65. Pre-treatment of lymphoma cells with the protein synthesis inhibitor cycloheximide (CHX) completely blocked bortezomib-induced up regulation of phosphorylated NF-κB and the levels of NF-κB were also reduced (Figure 6A). Bortezomib-mediated up-regulation of phosphorylated I-κBα was completely diminished by pre-treatment with CHX. The levels of the un-phosphorylated form of I-κBα were also remarkably reduced by CHX (Figure 6A). This suggests that *de novo* synthesized NF-κB and I-κBα may be more susceptible to IKK-mediated phosphorylation. The anti-apoptotic BCL-2 family proteins are also targets of NF-κB. As a result of NF-κB activation, MCL-1, BCL-XL and BCL-2 were up-regulated by bortezomib and increased expression of these proteins was partially inhibited when protein synthesis was blocked (Figure 6B). Protein levels of MCL-1, BCL-XL and BCL-2 were not increased while cells were cultured up to 18 hours without treatment (Figure S6). Other NF-κB target proteins, such as IL-6 and FLIP, were also up-regulated by treatment with bortezomib (Figure 6C). These results indicate that treatment with bortezomib induced *de-novo* protein synthesis and this is associated with NF-κB activation.

### Blocking autophagy potentiates proteasome inhibitor-mediated cell death

**Figure 7:**
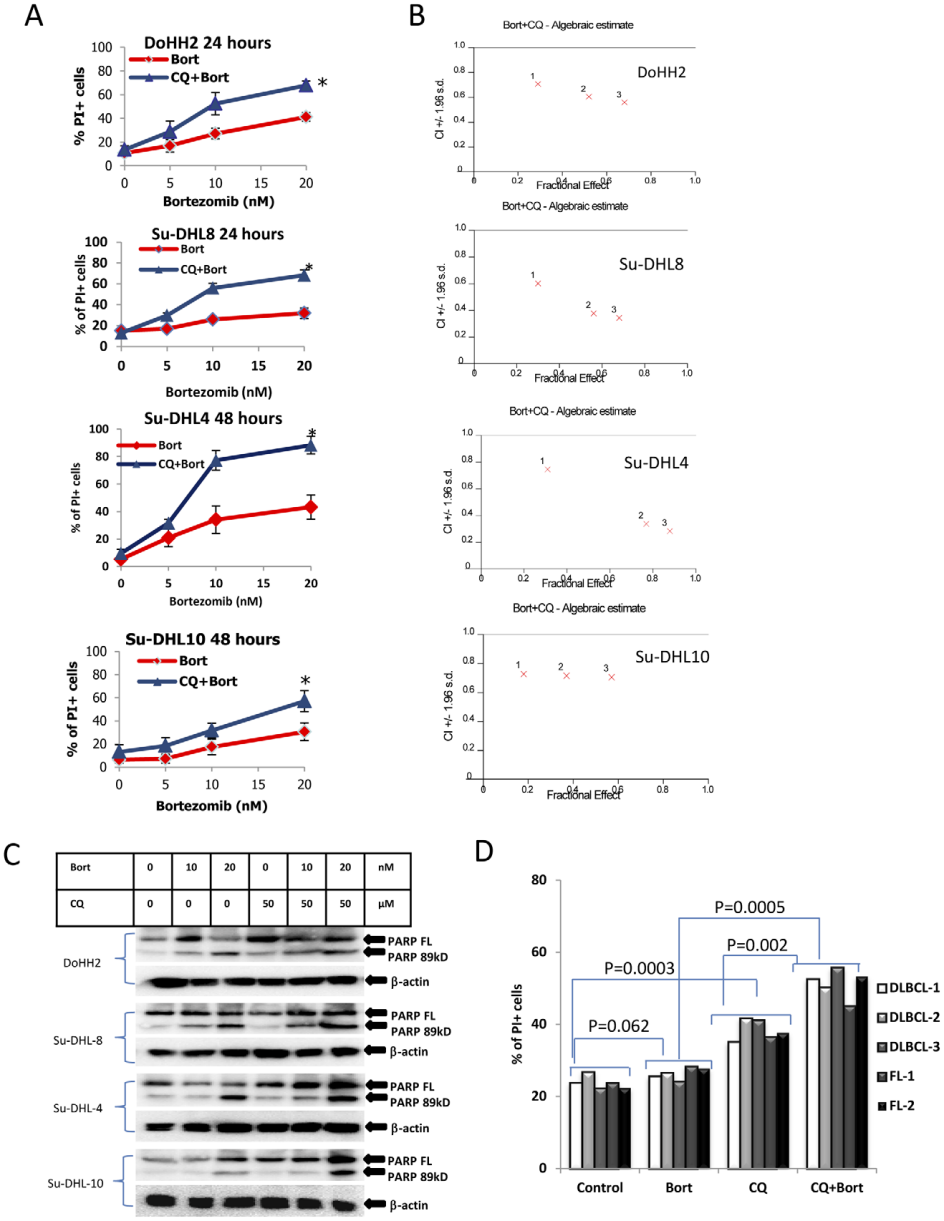
Synergistic effect of CQ on bortezomib-induced cell death. Cells were pre-treated with 50 µM CQ for I hour and then treated with bortezomib for 24 to 48 hours. (A) Determining cell death by flow cytometry. After treatment, cells were stained with 10 µg/ml PI and PI positive cells were counted as died cells. Data represent mean ± SD from 3 independent experiments. Asterisk (*) indicates P<0.01, significantly increased sensitivity analyzed by ANOVA. (B) Synergistic effect of CQ on bortezomib. Synergy was analyzed by CalcuSyn and synergy level was setup lower than 0.9. (C) PARP cleavage. PARP cleavage was analyzed by Western blotting using polyclonal anti-PARP antibody (1∶500). Lower bands (89 kD) indicate cleaved PARP. (D) Cell death in primary FL and DLBCL cells. Primary cells from liquid nitrogen were cultured in fresh medium for 3 hours. Cells were pre-treated with 30 µM CQ for 1 hour and then 20 nM bortezomib for 24 hours. Cell death was determined by flow cytometry. The significantly increased killing was analyzed by *t*-test.

To test whether autophagy acts as a resistant factor with this treatment, bortezomib-induced cell death was determined in the presence or absence of CQ. Treatment with CQ up to 50 µM did not induce significant cell death (Figure S7). Bortezomib-induced cell death was significantly increased (P<0.01) when combined with CQ. The maximum cell death-induced by CQ and bortezomib was reached in 24 hours for the bortezomib-sensitive cell lines, DoHH2 and Su-DHL8 and in 48 hours for the resistant cell lines, Su-DHL4 and Su-DHL10 (Figure 7A). The synergistic effect of CQ on bortezomib-induced cell death was analyzed using the CalcuSyn software [27]. CI values were all lower than 0.9, indicating synergy (Figure 7B). This showed that combination with CQ has a great potential to increase the sensitivity of DLBCL cell lines to bortezomib-induced cell death. The sensitization effect of CQ on bortezomib-induced apoptosis was determined by increased caspase-3 activity (Figure S8) and increased PARP cleavage (Figure 7C). We next tested whether inhibition of autophagy could overcome resistance of primary lymphoma cells to bortezomib-induced cell death. When FL and DLBCL primary tumor cells were treated with 20 nM bortezomib with or without 30 µM CQ, both FL and DLBCL primary cells were resistant to bortezomib (P = 0.062) but sensitive to CQ-induced cell death (P = 0.0003). The combination of CQ and bortezomib significantly sensitized lymphoma cells to treatment compared with using bortezomib (P = 0.0005) or CQ alone (P = 0.002) (Figure 7D). These data confirmed that inhibition of autophagy by CQ significantly synergized with bortezomib-induced lymphoma cell death by apoptosis.

## Discussion

This study demonstrated that treatment of DLBCL cells with bortezomib induces autophagy which eliminates ubiquitinated I-κBα. I-κBα is an inhibitor of NF-κB and sufficient levels of I-κBα protein are essential to keep NF-κB in the cytosol, preventing its transcriptional activity in the nucleus. Decreased levels of I-κBα could lead to NF-κB activation and cause further resistance of cancer cells to treatment [16], [38]. We demonstrate that blocking autophagy by CQ increased complex formation of I-κBα with NF-κB in the cytosol and prevented bortezomib-induced NF-κB activation.

Bortezomib is the first proteasome inhibitor and has been approved to treat relapsed multiple myeloma and mantle cell lymphoma [9], [39], [40]. A main anti-cancer effect of bortezomib is inhibition of NF-κB activation induced by TNF-α or TRAIL via blockade of I-κα degradation [2], [3]. Recent studies showed that constitutive NF-κB activity and microenvironment induced NF-κB activation in cancer cells can cause bortezomib-resistance [41], [42]. Of note, it has been recently shown that bortezomib does not prevent I-κBα degradation; in fact, it causes I-κBα degradation in multiple myeloma cells. This suggests that bortezomib-induced I-κBα degradation is not occurring through the UPS pathway [16].

Previous studies showed that ABC-DLBCL cell lines (OCI-Ly3 and OCI-Ly10) had high nuclear NF-κB DNA binding activity, constitutive I-κB kinase (IKK) activity and rapid I-κBα degradation that was not seen in GCB-DLBCL cell lines, such as Su-DHL-4. Abrogation of NF-κB activity by transduction of domain negative form of IKKβ was toxic to ABC-DLBCL cell but not to GCB-DLBCL cells, suggesting ABC type DLBCL cells rely on NF-κB for survival (19). Although ABC-DLBCL has constitutively activated NF-κB [19], [20], both GCB and ABC-DLBCL cells are resistant to bortezomib. We found that the sensitivity of DLBCL cells to bortezomib-induced apoptosis is independent of their types, as the Su-DHL-8 cell line (ABC-DLBCL) has constitutively activated NF-κB but is relatively sensitive to bortezomib compared with Su-DHL-4 and Su-DHL-10 (GCB-DLBCL). The differential sensitivities of these cells to bortezomib are more or less dependent on their constitutive ratios of BCL-2/Bax (results not shown). Our previous study showed that Bax is a short-lived protein and treatment with bortezomib leads to Bax accumulation in primary CLL cells and DLBCL cell lines [6]. We therefore reasoned that I-κBα, also a short-lived protein, could be accumulated in response to bortezomib. However, our results demonstrated that treatment with bortezomib induced I-κBα degradation in DLBCL cell lines as well as in primary FL and DLBCL cells, in agreement with the finding by Hideshima and colleagues in multiple myeloma cells [16].

Treatment with bortezomib induced autophagy in DLBCL cells. This is shown by accumulation of the autophagy marker LC3-II in parallel with I-κBα degradation. Autophagy and UPS are the major routes for intracellular protein degradation and the two pathways have previously been thought to be largely distinct [14]. UPS degrades short-lived proteins such as I-κBα, Bax and p53 while autophagy is a process to degrade long-lived proteins and organelles [43]. However, the short lived protein p53 accumulates when autophagy is inhibited [14], suggesting that the two degradation systems collaborate and compromise each other to remove unwanted proteins. We now show that treatment with bortezomib leads to accumulation of ubiquitinated proteins and CHOP, indicating that proteasome inhibition causes ER stress and that ubiquitin/ubiquitinated proteins translocate to p62 rich loci to form larger aggregates. Co-staining with p62 and LC3-II showed that these aggregates also contain LC3-II. p62 has multi-functional domains which can bind to ubiquitinated proteins and LC3 simultaneously, leading to ubiquitinated proteins being degraded by autophagy [31]. I-κBα co-localization and interaction with p62 was found before and after treatment with bortezomib. I-κBα staining and binding became weaker after treatment, consistent with the observed reduced levels of I-κBα shown by Western blotting. This suggests that I-κBα may be degraded by a route which is independent of the proteasome. It has been shown recently that proteasome inhibition induces accumulation of both ubiquitinated and non-ubiquitinated misfolded proteins in the aggresome, so that degradation by autophagy is not dependent on substrate ubiquitination [44]. However, I-κBα ubiquitination was clearly induced by bortezomib and was associated with I-κBα phosphorylation. It is unknown whether bortezomib-induced formation of p62/ubiquitin aggregates constitutes an aggresome and whether autophagy is triggered by aggresome formation.

The autophagy inhibitor CQ potentiates chemotherapeutic drug-induced apoptosis in lymphoma cells [45]. Inhibition of autophagy by CQ alone prevents lymphomagenesis in murine models [22]. We reasoned that I-κBα may be selectively degraded by autophagy after recruited by p62. The autophagy inhibitor CQ alone induced accumulation of ubiquitinated proteins, including I-κBα. However, phosphorylated I-κBα was not significantly up-regulated by CQ, indicating that CQ does not have effects upstream of I-κBα phosphorylation. In the presence of bortezomib, CQ prevented bortezomib-induced I-κBα degradation but not I-κBα phosphorylation.

Using the ABC-DLBCL cell line Su-DHL8, we confirmed that constitutively activated nuclear NF-κB was detected in un-stimulated cells. Bortezomib-induced I-κBα degradation decreased its complex formation with NF-κB and increased NF-κB nuclear translocation and therefore its DNA binding activity. Blocking autophagy by CQ sustained the levels of I-κBα protein and its binding to NF-κB and inhibited bortezomib-induced NF-κB activation. As a result of NF-κB activation, the increased transcriptional activity leads to up-regulation of anti-apoptotic proteins. This may induce lymphoma cells resistant to further treatment.

Inhibition of autophagy by CQ greatly sensitized DLBCL cells to proteasome inhibitor-induced cell death, in keeping with results in colon and ovarian cancer cells [13]. Interestingly, the most resistant cell line Su-DHL10 which lacks Bax/Bak proteins [23], underwent 60% cell death in response to the combined treatment with both CQ and bortezomib (at 20 nM), indicating that blocking both autophagy and proteasome degradation is a potent therapeutic strategy to kill apoptosis-resistant lymphoma cells. Although CQ sensitized DLBCL cells to proteasome inhibitor-mediated cell death, the differential sensitivities of these DLBCL cells to proteasome inhibitor was still persistent in the presence of CQ, suggesting that CQ and bortezomib-induced cell death is mitochondria-dependent.

In summary, here we report, for the first time, that bortezomib-induced I-κBα degradation in DLBCL cells may be due to activated autophagy. Autophagy plays a protective role in proteasome inhibitor-induced cell death by eliminating cytotoxic ubiquitinated proteins. It is well known that unbalanced expression of BCL-2 family proteins confers differential sensitivities of cancer cells to chemotherapeutic drugs, including bortezomib. Autophagy is another resistant factor which diminishes therapeutic effect in both sensitive and resistant lymphoma cells. Elimination of I-κBα by autophagy induces NF-κB activation and promotes cell survival. We therefore propose that blocking proteasome and autophagy simultaneously can overcome resistance of DLBCL cells to bortezomib-induced cell death.

## Supporting Information

Figure S1 **Daunorubicin uptake.** Cells in culture medium were subjected to the flow cytometry and the base line of the red fluorescence was obtained by monitoring unstained cells for a few seconds. After adding 100 µg/ml daunorubincin, the red fluorescent intensity was continuously monitored for 102 seconds on the FL3-H channel.
(TIF)

Figure S2 **Correlation of bortezomib-induced accumulation of LC3-II and poly-ubiquitin.** The levels of accumulated poly-ubiquitin and LC3-II were analyzed by densitometry and calculated as ratio of Ub/actin and LC3-II/actin, respectively. Correlation between two levels of two proteins was analyzed by Correlation (Prism).
(TIF)

Figure S3 **Bortezomib and CQ induced co-localization and aggregation of p62 and ubiquitin.** Su-DHL10 cells were treated with 10 nM Bortezomib or/and 50 µM CQ for 24 hours. After fix/permeabilization, cells on slides were co-stained with a polyclonal anti-p62 antibody (green) and a monoclonal anti-ubiquitin antibody (red).
(TIF)

Figure S4 **Bortezomib and CQ induced co-localization and aggregation of p62 and LC3.** Su-DHL10 cells were treated with 10 nM Bortezomib, or/and 50 µM CQ for 24 hours. After fix/permeabilization, cells on slides were co-stained with a polyclonal anti-LC3B antibody (green) and a monoclonal anti-p62 antibody (red).
(TIF)

Figure S5 **Co-localization of p62 and I-κBα.** Su-DHL8 cells were pre-incubated with or without 50 µM CQ for 1 hour and then treated with 20 nM bortezomib for 24 hours. Cells on slides were co-stained with polyclonal anti-I-κBα antibody (1∶20) and monoclonal anti-p62 antibody (1∶20) and then probed with Alexa Fluor anti-mouse IgG 546 (1∶50 dilution) and anti-rabbit IgG-488 (1∶100 dilution). Arrows indicate p62-I-κBα aggregates.
(TIF)

Figure S6 **Expression of MCL-1, Bcl-XL and Bcl-2 in the absence of treatment.** Su-DHL8 cells were cultured up to 18 hours. Polyclonal anti-Mcl-1 (1∶1000) and Bcl-XL (1∶200) antibodies, and monoclonal anti-Bcl-2 antibody (1∶200) were used for Western blotting.
(TIF)

Figure S7 **CQ induced cell death.** Cells were treated with 50 µM CQ for 24 hours. Cells were stained with PI and then cell death was accessed by flow cytometry. Data presented are mean ± SD from 3 independent experiments.
(TIF)

Figure S8 **Effect of CQ on bortezomib-induced caspase activation.** Cells were pre-treated with 50 µM CQ for 1 hour and then treated with bortezomib for 16 to 24 hours. Data are mean ± SD from 3 independent experiments.
(TIF)
