# Supplementary figures and images for: Blocking Autophagy Prevents Bortezomib-Induced NF-κB Activation by Reducing I-κBα Degradation in Lymphoma Cells
Source: PLoS One. 2012 Feb 29;7(2):e32584. doi: 10.1371/journal.pone.0032584 (PMC3290566; doi:10.1371/journal.pone.0032584)

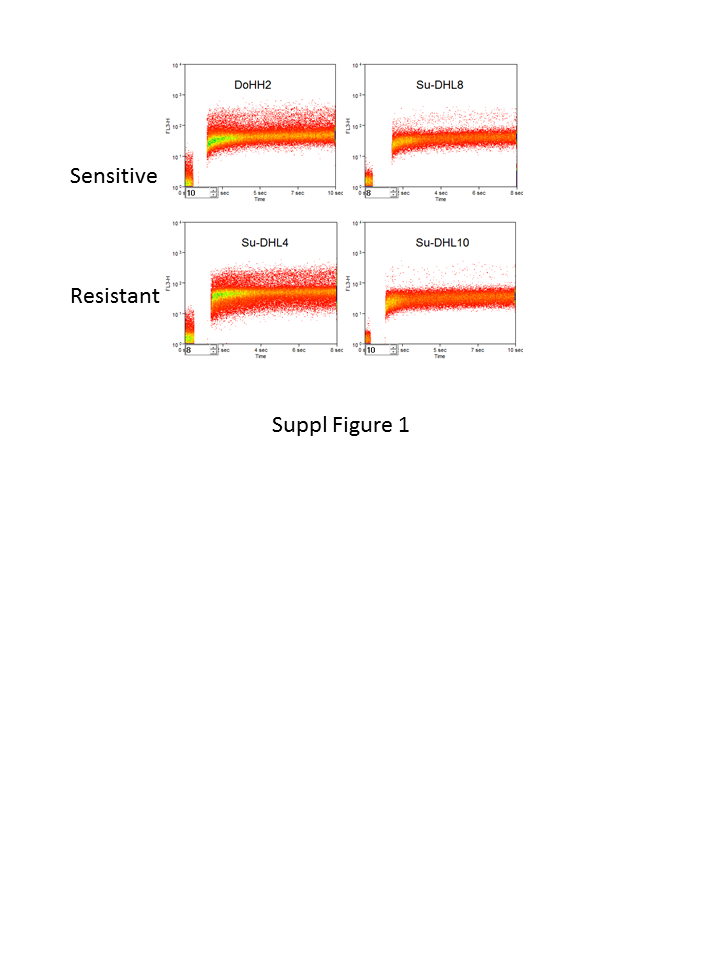

Supplement: Figure S1 — Daunorubicin uptake. Cells in culture medium were subjected to the flow cytometry and the base line of the red fluorescence was obtained by monitoring unstained cells for a few seconds. After adding 100 µg/ml daunorubincin, the red fluorescent intensity was continuously monitored for 102 seconds on the FL3-H channel. (TIF) [file pone.0032584.s001.tif]

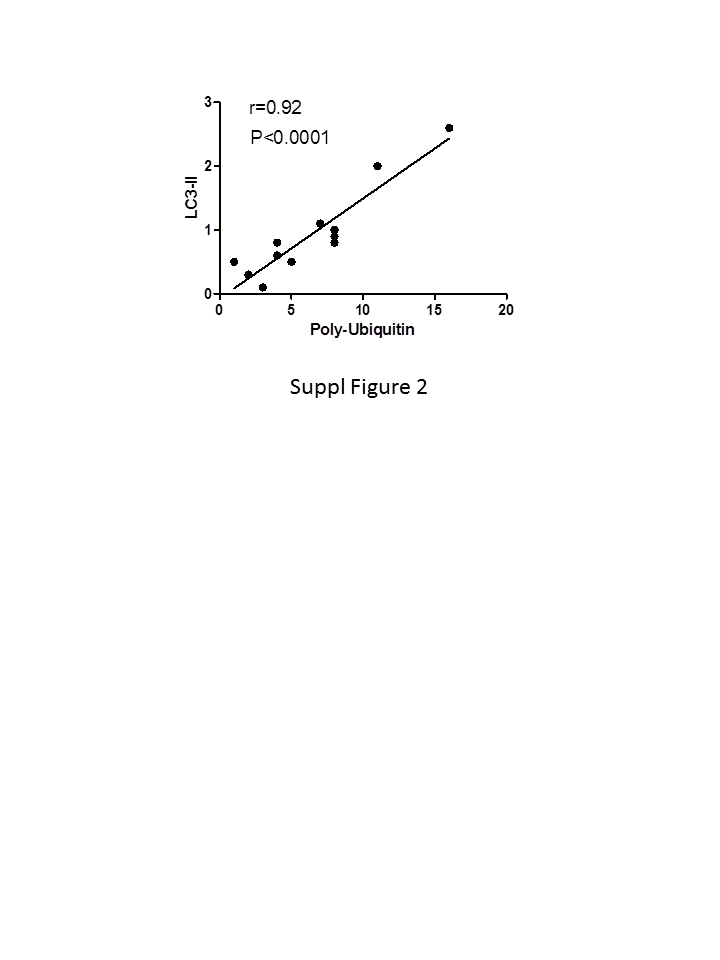

Supplement: Figure S2 — Correlation of bortezomib-induced accumulation of LC3-II and poly-ubiquitin. The levels of accumulated poly-ubiquitin and LC3-II were analyzed by densitometry and calculated as ratio of Ub/actin and LC3-II/actin, respectively. Correlation between two levels of two proteins was analyzed by Correlation (Prism). (TIF) [file pone.0032584.s002.tif]

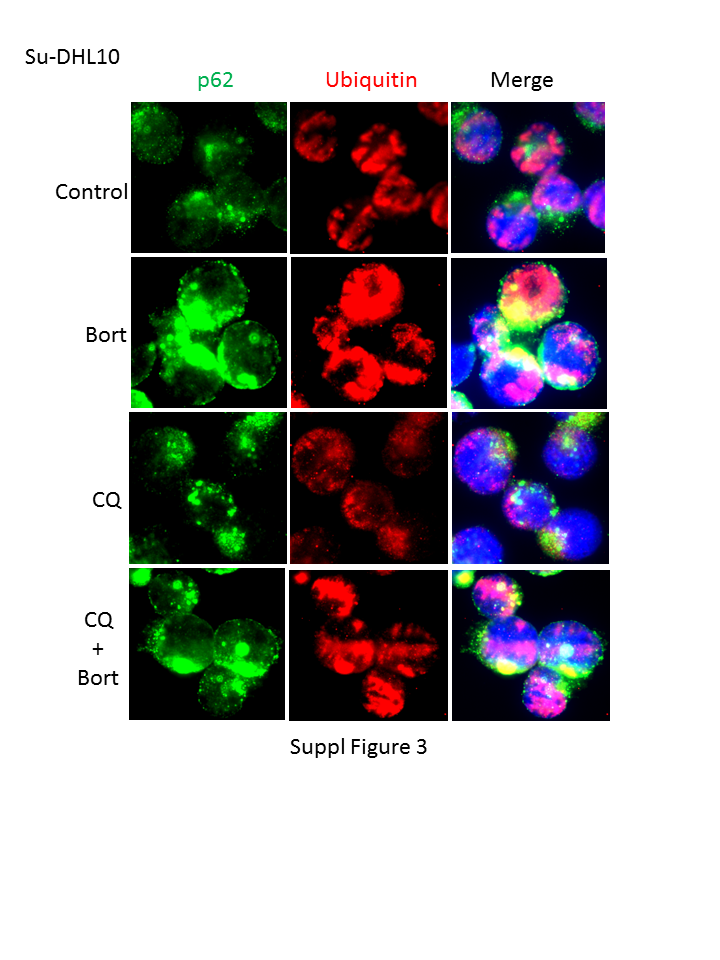

Supplement: Figure S3 — Bortezomib and CQ induced co-localization and aggregation of p62 and ubiquitin. Su-DHL10 cells were treated with 10 nM Bortezomib or/and 50 µM CQ for 24 hours. After fix/permeabilization, cells on slides were co-stained with a polyclonal anti-p62 antibody (green) and a monoclonal anti-ubiquitin antibody (red). (TIF) [file pone.0032584.s003.tif]

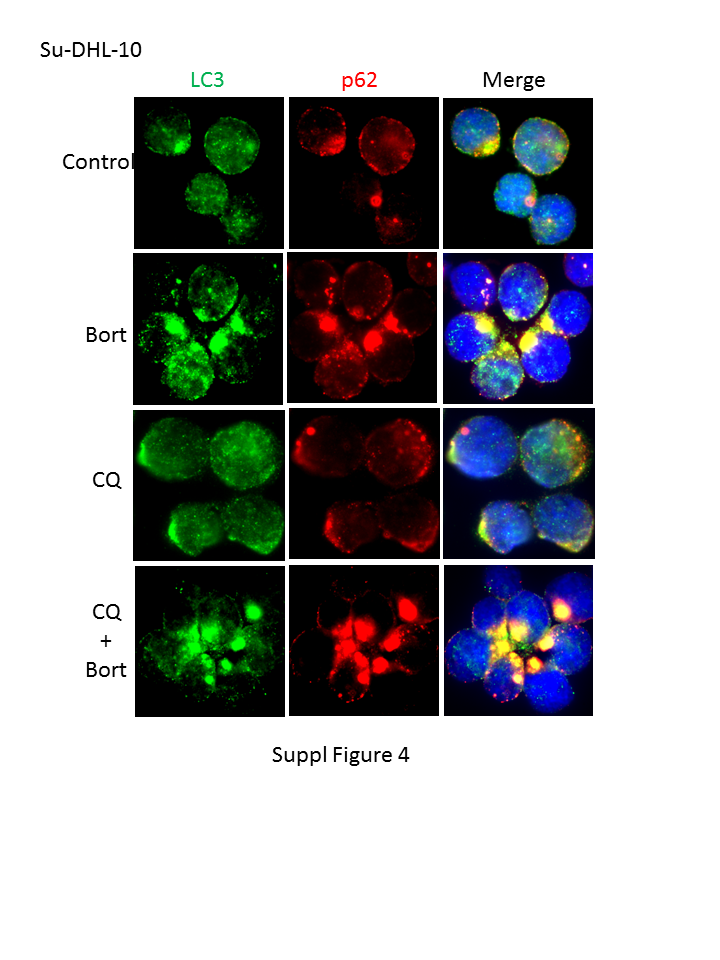

Supplement: Figure S4 — Bortezomib and CQ induced co-localization and aggregation of p62 and LC3. Su-DHL10 cells were treated with 10 nM Bortezomib, or/and 50 µM CQ for 24 hours. After fix/permeabilization, cells on slides were co-stained with a polyclonal anti-LC3B antibody (green) and a monoclonal anti-p62 antibody (red). (TIF) [file pone.0032584.s004.tif]

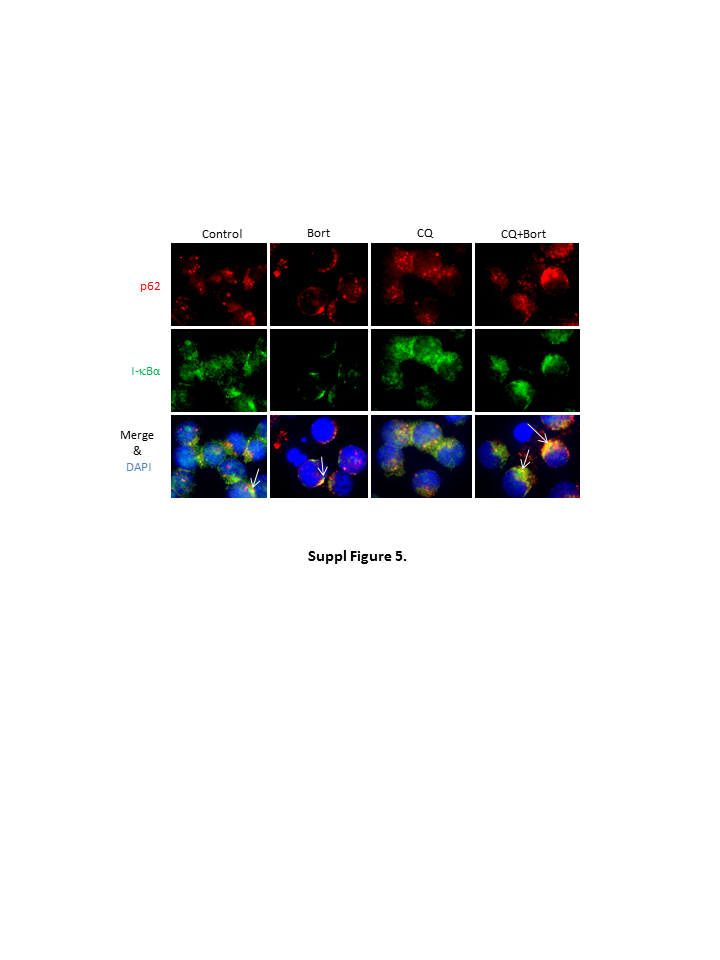

Supplement: Figure S5 — Co-localization of p62 and I-κBα. Su-DHL8 cells were pre-incubated with or without 50 µM CQ for 1 hour and then treated with 20 nM bortezomib for 24 hours. Cells on slides were co-stained with polyclonal anti-I-κBα antibody (1∶20) and monoclonal anti-p62 antibody (1∶20) and then probed with Alexa Fluor anti-mouse IgG 546 (1∶50 dilution) and anti-rabbit IgG-488 (1∶100 dilution). Arrows indicate p62-I-κBα aggregates. (TIF) [file pone.0032584.s005.tif]

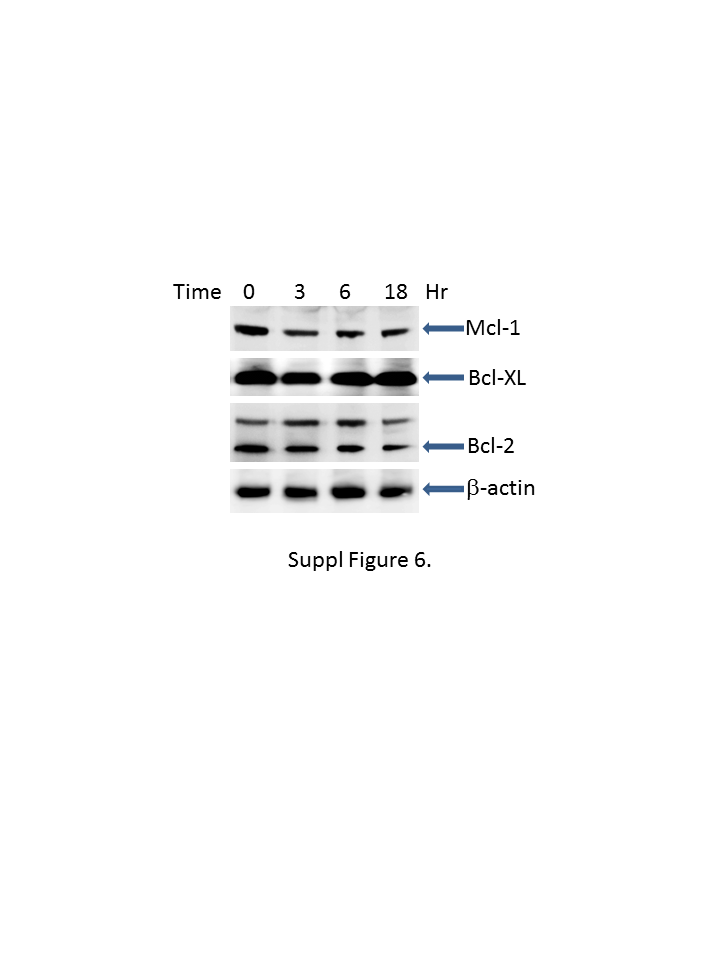

Supplement: Figure S6 — Expression of MCL-1, Bcl-XL and Bcl-2 in the absence of treatment. Su-DHL8 cells were cultured up to 18 hours. Polyclonal anti-Mcl-1 (1∶1000) and Bcl-XL (1∶200) antibodies, and monoclonal anti-Bcl-2 antibody (1∶200) were used for Western blotting. (TIF) [file pone.0032584.s006.tif]

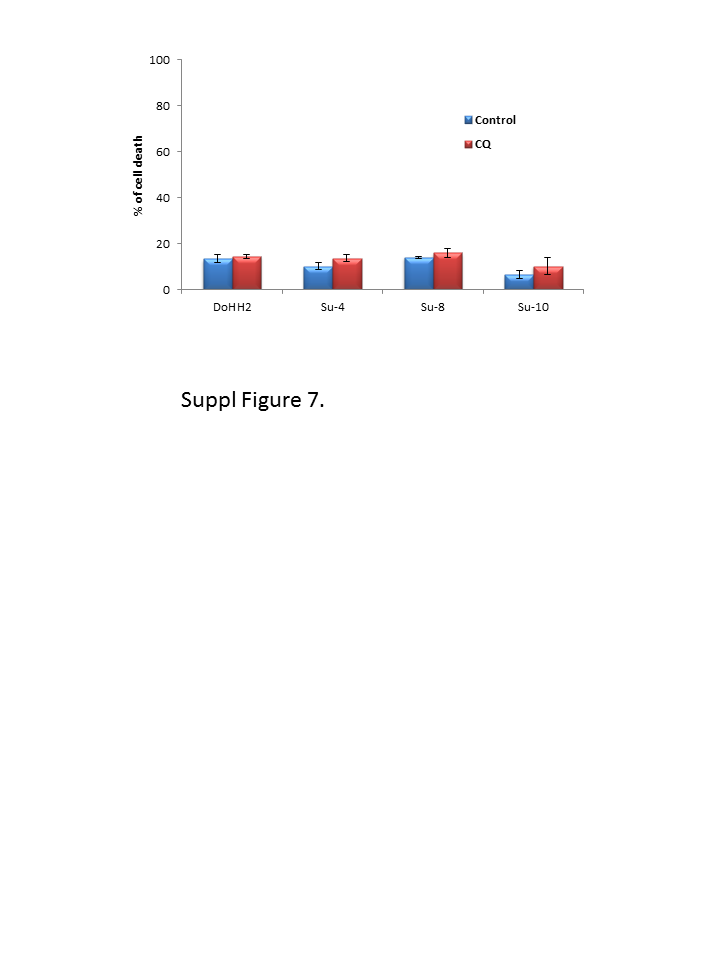

Supplement: Figure S7 — CQ induced cell death. Cells were treated with 50 µM CQ for 24 hours. Cells were stained with PI and then cell death was accessed by flow cytometry. Data presented are mean ± SD from 3 independent experiments. (TIF) [file pone.0032584.s007.tif]

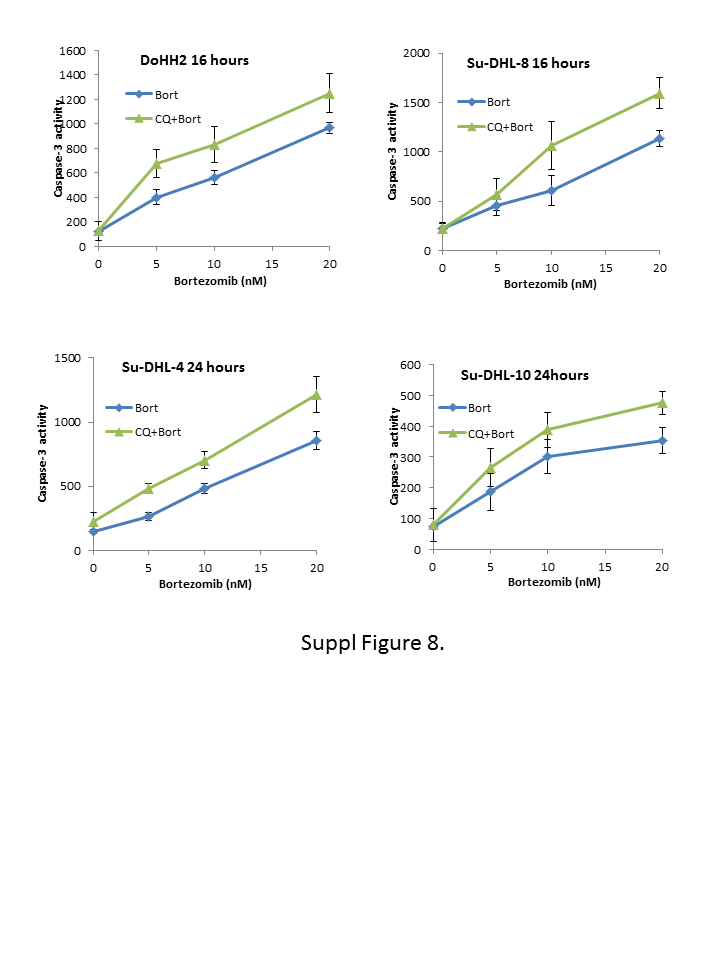

Supplement: Figure S8 — Effect of CQ on bortezomib-induced caspase activation. Cells were pre-treated with 50 µM CQ for 1 hour and then treated with bortezomib for 16 to 24 hours. Data are mean ± SD from 3 independent experiments. (TIF) [file pone.0032584.s008.tif]
